# Selection of patients with pancreatic adenocarcinoma who may benefit from radiotherapy

**DOI:** 10.1186/s13014-023-02328-y

**Published:** 2023-08-18

**Authors:** I-Shiow Jan, Hui Ju Ch’ang

**Affiliations:** 1 Department of Laboratory Medicine, College of Medicine, National Taiwan University Hospital, National Taiwan University, Taipei, Taiwan; 2 National Institute of Cancer Research, National Health Research Institutes, Miaoli, Taiwan; 3 Program for Cancer Biology and Drug Discovery, College of Medical Science and Technology, Taipei Medical University, Taipei, Taiwan; 4 Department of Radiation Oncology, Taipei Medical University, Taipei, Taiwan; 5 Department of Oncology, School of Medicine, College of Medicine, National Cheng Kung University, Tainan, Taiwan

**Keywords:** Pancreatic adenocarcinoma, Radiotherapy, Biomarker, Radiomic

## Abstract

Despite combination chemotherapy demonstrating a positive effect on survival, the clinical outcomes of pancreatic adenocarcinoma (PDAC) remain poor. Radiotherapy was previously a component of the curative treatment of PDAC. Advances in imaging and computer sciences have enabled the prescription of higher dosage of radiation focused on tumours with minimal toxicity to normal tissue. However, the role of radiotherapy has not been established in the curative treatment of localized PDAC because of the conflicting results from large prospective trials. Most studies have demonstrated improved locoregional control but no survival benefit from additional chemoradiotherapy (CRT) in addition to chemotherapy for resectable, borderline or locally advanced PDAC. The improved locoregional control enabled by CRT does not cause extended survival because of rapid distant progression in a significant proportion of patients with PDAC. Several single-institute studies of prescribing intensive chemotherapy with modern ablative radiotherapy for locally advanced PDAC have demonstrated extended survival with an acceptable safety profile. In an analysis after long-term follow-up, the PREOPANC study demonstrated a survival benefit from neoadjuvant gemcitabine-based CRT in resected PDAC relative to upfront surgery followed by adjuvant gemcitabine only. These observations indicated that the role of radiotherapy in PDAC should be evaluated in a subgroup of patients without rapid distant progression because systemic therapy for PDAC remains underdeveloped. We reviewed critical imaging, tissue, liquid and clinical biomarkers to differentiate the heterogeneous biologic spectra of patients with PDAC to identify those who may benefit the most from local radiotherapy. Exclusion of patients with localised PDAC who develop distant progression in a short time and undergo extended upfront chemotherapy for over 4 months may enable the identification of a survival benefit of local radiotherapy. Though promising, the effectiveness of biomarkers must be validated in a multi-institutional prospective study of patients with PDAC receiving CRT or not receiving CRT.

## Background

### No evidence of survival benefit from radiotherapy as curative treatment for PDAC

Pancreatic ductal adenocarcinoma (PDAC) is one of the most severe malignancies among all solid tumours, with a 5-year survival rate of less than 10% [1, 2]. Most patients with PDAC present with locally advanced pancreatic cancer (LAPC) or metastatic disease that is not suitable for resection [3]. Chemotherapy, radiotherapy, and modern targeted, immunologic therapy exhibit limited efficacy in treating PDAC. Therefore, patients with PDAC usually experience rapid recurrence in the form of locally destructive diseases or distant metastasis [4, 5].

The development of combination chemotherapy consisting of (modified) leucovorin calcium (**fol**inic acid), **f**luorouracil, **irin**otecan hydrochloride, **ox**aliplatin (FOLFIRINOX) [6, 7], and gemcitabine plus nab-paclitaxel (GEM-Nab) [8] has resulted in superior tumour response and survival compared with chemotherapy using single- agent GEM or 5-fluorouracil (5FU) in patients with metastatic or unresectable PDAC. Prospective randomized trials have demonstrated the overall survival (OS) benefit of adjuvant chemotherapy using FOLFIRINOX (54.4 vs. 35.0 months, *p* = 0.003) [9], GEM plus capecitabine (GEM-Cape; 28.0 vs. 25.5 months, *p* = 0.032) [10], or GEM plus nab-paclitaxel (41.8 vs. 37.7 months, *p* = 0.009) [11] compared with using single-agent GEM to treat resected PDAC. For borderline resectable PDAC, neoadjuavant chemotherapy achieves a higher R0 resection rate and survival than does upfront surgery [12–14]. A meta-analysis of seven trials with 938 patients revealed significantly improved OS using neoadjuvant therapy (29 vs. 19 months, *p* = 0.001), especially among patients with borderline resectable PDAC (*p* = 0.004) [15].

Unlike that of chemotherapy for PDAC, the efficacy of radiotherapy as an adjuvant or curative treatment for PDAC is limited. The results of the European Study Group for Pancreatic Cancer-1 (ESPAC-1) trial led to the omission of radiotherapy from most European adjuvant trials involving resectable PDAC [16]. We conducted a prospective randomised study to evaluate chemo-radiotherapy (CRT) with adjuvant 6-month GEM. The results indicated improved local control (loco-regional recurrence rate of GEM vs. GEM-CRT arms: 54.1% vs. 38.4%, *p* = 0.056) but no survival benefit (median OS of GEM vs. GEM-CRT: 23.5 vs. 21.5 months, *p* = 0.73 ) from administering additional CRT to patients with curatively resected PDAC [17]. The results of the Radiation Therapy Oncology Group (RTOG) 0848 study evaluating adjuvant CRT in resected PDAC after adjuvant GEM are highly anticipated [18]. However, the impact of RTOG 0848 may be less relevant because FOLFIRINOX and GEM-Cape have become the standard of care for adjuvant chemotherapy [9, 10]. For borderline resectable PDAC, the PREOPANC-1 study [13, 14] demonstrated long-term survival improvement (median OS: 15.7 vs. 14.3 months, *p* = 0.025; 5-year survival rate: 20.5% vs. 6.5%) with neoadjuvant GEM-based CRT and improved loco-regional control (*p* = 0.004) compared with adjuvant GEM alone. The ESPAC-5 [19] and A021501 [20] studies have demonstrated extended survival with neoadjuvant chemotherapy especially using FOLFIRINOX in ESPAC-5 (1-year survival rate: 84% vs. 39% for immediate surgery, *p* = 0.0028). Despite the high R0 resection and pathologic complete remission rate, neoadjuvant radiotherapy was not associated with favourable survival in either study. For LAPC, the LAP07 study [21] identified better local control (46% vs. 32%, *p* = 0.03) but no survival benefit (11.9 months vs. 13.6 months, *p* = 0.09) from the addition of CRT after induction GEM. These results conflict with the report from the Eastern Cooperative Oncology Group trial, which indicated a survival benefit from upfront GEM-based CRT compared with GEM alone (11.1 vs. 9.2 months, *p* = 0.017) [22]. The conflicting results of the randomized studies concerning borderline resectable and locally advanced PDAC imply a narrow therapeutic window associated with radiotherapy.

### Reasons of continued evaluation of radiotherapy for curative PDAC treatment

The role of CRT has been questioned because of controversial clinical trial results. However, CRT remains under careful consideration for PDAC for several reasons: First, the survival outcomes of PDAC remain inferior compared to those of other solid tumours. Novel therapeutic options and modern techniques including stereotactic body radiotherapy (SBRT), magnetic resonance (MR) imaging guided radiotherapy and proton therapy enabled highly conformal and tolerable radiation to be given with solutions for respiratory motion and reduced toxicity to the gastrointestinal area [23, 24]. The Massachusetts General Hospital group demonstrated total neoadjuvant therapy with eight cycles of FOLFIRINOX and losartan, an inhibitor of thrombospondin-1 mediated activation of latent tumour growth factor β (TGFβ), followed by a short or long course of modern radiotherapy for 49 patients with LAPC resulted in a high rate of down-staging and R0 resection in 61% of patients, with a median progression-free survival (PFS) and OS of 17.5 and 31.4 months, respectively [25]. Ablative radiotherapy of 75 Gy in 25 fractions was administered to 119 patients with inoperable PDAC following multiagent induction chemotherapy at Memorial Sloan Kettering Cancer Center. The retrospective analysis revealed safe and durable local control with a median OS of 26.8 months [26]. These studies may influence and inspire current standard approaches. Second, the margin positivity rate and locoregional recurrence rate are high in PDAC, despite radical surgery and intensive systemic chemotherapy [9, 10, 27]. A rapid autopsy study indicated that one-third of patients with PDAC die from local destructive disease without widespread distant metastasis [28]. The efficacy of locoregional control and palliation by radiotherapy has been demonstrated in most studies of PDAC. Jolissaint et al. compared the clinical outcomes of patients with PDAC receiving ablative radiotherapy (n = 104) or surgical resection (n = 105). Despite a selection bias favouring the surgical group, the incidence of locoregional recurrence was similar (16% vs. 21%, *p* = 0.252) [29]. The excellent locoregional outcomes achieved using modern radiotherapy should be integrated into multimodality treatment of PDAC. Third, the survival benefit of CRT has been demonstrated after exclusion of patients with PDAC with early progression. In the PREOPANC study [14], a significant survival benefit was demonstrated for CRT after long term follow-up (*p* = 0.025). The steep initial slope of the survival curve, representing early progression, starts to bend and clearly separate from that of patients not receiving CRT after a year from diagnosis, indicating a small difference in median survival (1.4months; 15.7 vs. 14.3 months) between the groups; 5- year survival exhibited a 14% difference (20.5% vs. 6.5%). These results are consistent with the general consensus to prescribe CRT after initial systemic treatment. Accordingly, selecting patients with PDAC with low risk of early disease progression is crucial to translate local control using CRT into a survival benefit.

This review highlights the role of biomarkers in predicting patients with PDAC with low risk of early progression and who are thus suitable for being considered for subsequent radiotherapy with or without concomitant chemotherapy. A biomarker is a characteristic that is objectively measured and evaluated as an indicator of normal biological processes, pathogenic processes or pharmacologic responses to therapeutic intervention [30].

## Potential biomarkers for identifying patients with PDAC suitable for radiotherapy

### Imaging biomarkers

Radiomics, refers to the extraction and analysis of numerous quantitative features from medical images, and it has shown early promise in the analysis of imaging features and in prognostic modeling and outcome analysis [31]. The baseline imaging textural profile of the tumour microenvironment, including vascularity and oxygenation, and tumor heterogeneity was correlated with pathologic and clinical outcomes in resected PDAC (Table 1). Radiomic features derived from textural signals and groupings of pixels of baseline contrast-enhanced computed tomography (CT) in resectable PDAC were demonstrated to predict OS after surgery [32]. The signal intensity multiplied by the contour volume of pancreas was inversely associated with the pathologic lymph node category and correlated with the OS and PFS of patients with resected PDAC [33]. A seven-feature radiomic signature of a contrast-enhanced CT simulation scan could predict locoregional recurrence in patients with PDAC receiving SBRT [34]. Blood perfusion of tumor from CT scans was correlated with fractional tumour cell death in PDAC. The normalised area under the enhancement curve (nAUC) was correlated with OS and response to CRT patients with borderline resectable PDAC and LAPC [35]. These studies demonstrated baseline CT to be a potential tool for predicting the clinical outcomes of PDAC. If further validated, the signature could be used to help select patients who may benefit from neoadjuvant or adjuvant CRT.

**Table 1:** Studies of potential radiomic biomarkers for PDAC patients considering radiotherapy

Study	Study type	Pt No.	PDAC stage	Image	Timing	Radiomic parameter	Endpoints	Conclusion	Significance
2019 Khalvati F [32]	Retrospective	98	Resectable	Baseline CT	Pre-op	Sum entropy, cluster tendency features	OS	May stratify patients for NAT or alternatives	HR 1.56, 1.35 p = 0.005; 0.022 for two readers
2022 Elsherif SB [33]	Retrospective	54	Resectable/ BR	Baseline Dual energy CT	Pre-NAT	∫ T (HU·mL) (PPP) = 507.85	OS, PFS	Predict pathologic lymph node status	P = 0.006 P = 0.001
2020 Parr E [34]	Retrospective	74	Localized	Contrast- enhance CT	RT planning CT	6 or 7 radio-genomic features	OS Loco-regional recurrence	OS and local recurrence better predict by radiomic features than clinical features	6 features for OS: p < 0.0001; 7 features for loco-regional recurrence: p < 0.0001
2022 Wang CX [35]	Retrospective	297	BR & LA	Pre, arterial, venous phase contrast-enhanced CT	Before CRT	Normalized area under the curve (nAUC)	OS	nAUC, correspond to tumor cell death validated by histo-pathology, predict OS	P < 0.0001
2022 Koay EJ [36]	Prospective Nab-Cape with concomitant RT after ICT	23	BR & LA	Baseline (post- ICT) and follow-up (post-CRT) CT	Post-ICT, post-CRT	Type I (remains or sharper) and II (blurring) interface response	OS, PFS	Type I interface response associate with better OS and PFS	p = 0.004, p = 0.03
2022 Rossi G [37]	Retrospective	71	LA	Contrast-enhanced planning CT for RT	Post-ICT	4/1655 radiomic features	Resectability	4 features-model predict resectability after NAT	AUC: 0.944
2019 Cozzi L [38]	Retrospective	100	LA for SBRT	Contrast free planning CT	Before SBRT	9 features for OS 4 features for local control	OS Local control	Low risk group had OS 15.1 and local control 28.6 months	P = 0.05 P = 0.004
2018 Cheng Z [39]	Retrospective	191	BR & LA	Planning CT for SBRT	Before SBRT	Overlap-volume histograms of GTV to key arterial structure	Margin negative resection	Tumor involved > 1 cm key arterial structure, less likely to have margin negative resection (23% vs. 77%)	P < 0.01
2019 Nasief H [40]	Retrospective	24	Resectable or BR	Daily non-contrast CTs during CT-guided CRT	During CRT	73 Delta radiomic features/ >1300 radiomic features	Response and OS	Decreased delta radiomic features and CA19-9 predict better OS	P = 0.001 P = 0.031
2019 Yamamoto KN [41]	Retrospective	1089	LA	Three sequential CT during ICT	FFX, GEM or Gem + Nab	Time series tumor volume data derived Local/metastatic advancement index (LAI/MAI)	Primary tumor size, metastatic number, OS	1. RT after ICT improves OS in larger LAI 2. CRT leads to a significant survival benefit when FFX but not GEM or GEM + Nab	1.P = 0.0547,0.0429,0.0379 for FFX, Gem, Gem + Nab; 2. p = 0.008, 0.236, 0.253 for FFX, GEM, GEM + Nab
2018 Bali MA [42]	Prospective	24	PDAC receiving chemotherapy	DW-MR	Baseline, wk2, wk8 post-chemotherapy	ROI-ADC, DW-volume, diffusion parameters	Response	At wk2, 25th percentile of H-D and H-PF change correctly classified response in 20/24 pts; at wk8, DW-volume change correctly classified 22/24 pts	P = 0.003 P < 0.0001
2020 Itchins M [44]	Retrospective	115	Resectable & BR	PET	Baseline and pre-op	SUV(max)	OS	Pre-op SUV(max) < 5 after NAT predict improved OS (42.95 vs. 26.05 months)	P = 0.02
2022 Abdelrahman AM [45]	Retrospective	202	Resected BR/LA	PET	Before and after NAT	Metabolic response	Pathologic response, OS	Metabolic response predict pathologic response and OS	P < 0.001 P < 0.001
2021 Panda A [46]	Retrospective	44	BR & LA	PET	Before and after NAT	Complete metabolic response, mean change in SUVmax	Pathologic response OS	Change in SUVmax and complete metabolic response were associated with OS	P < 0.05
2021 Zimmermann C [47]	Prospective	25	Resectable BR & LA	PET/CT	Before and after NAT	Decreased SUVmax ≥ 30%	Response	Median SUVmax decreased after NAT (8.29 and 3.83)	p < 0.001
2017 Sakane M [48]	Retrospective	25	Resectable & BR	PET/CT	Before and after CRT	SUVpeak, MTV, TLG	Pathologic response	Higher post-CRT SUVpeak, positive MTV/TLG predict unfavorable pathologic effects of CRT	P = 0.013 P = 0.014

Pt: patient; Pre-op: preoperative; BR: borderline resectable; LA: locally advanced; NAT: neoadjuvant therapy; RT: radiotherapy; SBRT: stereotactic body radiotherapy; CRT: chemoradiotherapy; GTV: gross tumor volumne; ICT: induction chemotherapy; OS: overall survival; PFS: progression free survival; FFX: FOLFIRINOX; GEM: gemcitabine; Nab: nab-paclitaxel; Cape: capecitabine; ROI-ADC: regions-of-interest-apparent diffusion coefficent; DW-volume: diffusion weighted-volume; H-D: histogram D pure diffusion; H-PF: histogram perfusion fraction; SUV: standardized uptake value; TLG: total lesion glycolysis; MTV: metabolic tumor volume

CT imaging profiles after upfront chemotherapy for PDAC are associated with clinical outcomes. A more defined interface response of tumor post chemotherapy was associated with prolonged OS among patients with borderline resectable or locally advanced PDAC [36]. Four radiomic features from simulation CT scans were selected to construct a model to predict resectability in LAPC after neoadjuvant CRT [37]. Radiomic signatures indicating the relationship between tumours and key arteries from CT for radiotherapy treatment planning predicted local control, resectability and OS for borderline resectable and locally advanced PDAC cases after systemic chemotherapy [38, 39]. Patients’ longitudinal radiomic data progress throughout treatment (delta-radiomics) were able to help assess treatment response earlier and more reliably [40]. Yamamoto et al. established a logistic growth pattern of PDAC and defined the Local Advancement Index (LAI) to determine eventual primary tumour size and predict the number of metastases; a smaller LAI value indicates a larger metastatic burden. Radiotherapy after induction chemotherapy improved the survival of patients with larger LAI values [41]. The subgroup of patients with PDAC suitable for consolidative CRT after upfront or induction chemotherapy may be differentiated using potential radiomic parameters developed after chemotherapy.

Furthermore, diffusion-weighted MR quantitative metrics after chemotherapy were demonstrated to indicate response of patients with PDAC to chemotherapy [42]. Collagen molecular imaging using selective MR enhancement of fibrosis with CM-101, a type I collagen-targeted probe, revealed a robust fibrotic response after neoadjuvant therapy of FOLFIRINOX and correlated with improved survival in murine model of PDAC receiving CRT [43]. The preoperative uptake value of fluoro-deoxyglucose positron emission tomography (FDG-PET) and metabolic response to neoadjuvant therapy could predict the OS of patients with PDAC [44–48].

The ability of radiomic signatures to provide superior information for evidence-based clinical decision-making regarding PDAC is promising. To select patients who will benefit from radiotherapy, potential radiomic signatures should be explored in prospective clinical trials and validated through expansion of the available dataset, preferably in a multi-institutional study. Standardisation of radiomic signatures and imaging modalities to reduce inter-observer variability is also necessary.

### Histopathologic, liquid and clinical biomarkers

Molecular classifications of PDAC based on genomic, transcriptomic, proteomic and epigenetic data have provided considerable insights into the molecular heterogeneity and aggressive biology of PDAC [49]. Several potential biomarkers have been demonstrated to enable differentiation of the failure patterns in patients with PDAC. (Table 2) *SMAD4* gene status and expression have been highly correlated with radiosensitivity and the initial failure site of PDAC in clinical and preclinical studies [28, 50, 51]. In a phase II prospective study of 69 patients with LAPC, a local dominant pattern of progression was identified in patients with intact *SAMD4* and not in those with *SMAD4* loss (73% vs. 28%, *p* = 0.016) [52]. A retrospective study of 641 patients with resected PDAC demonstrated that inactivated *SMAD4* was strongly associated with metastatic recurrence (hazard ratio (HR) = 4.28, 95% CI = 2.75–6.68). Improved survival with additional radiotherapy was observed only in patients with PDAC with *SMAD4* expression (*p* = 0.002). The investigators concluded that patients with *SMAD4* expression benefit more from intensive local control [53]. Whittle et al. further demonstrated that heterozygous mutation of *SMAD4* attenuated the metastatic potential of PDAC and increased its proliferation. Loss of the heterozygosity of *SMAD4* restored metastatic competency and further increased proliferation– a highly lethal combination. The authors further demonstrated that *RUNX3* responded to and interacted with *SMAD4* status to regulate the balance between cancer cell division and dissemination, and they suggested that *RUNX3* and *SMAD4* levels can be used together to inform clinical decision-making for resectable PDAC [54]. Krüppel-like factor 10 (*KLF10*), a TGFβ early-response gene, has been demonstrated by investigators, including us, to contribute to PDAC radiosensitivity, epithelial - mesenchymal transition, and cancer stemness and progression [55–57]. We evaluated potential biomarkers including *SMAD4, RUNX3* and *KLF10* in tumour tissues from 111 patients with resected PDAC randomised to adjuvant GEM with or without CRT [58]. Loss of both *SMAD4* and *KLF10* expression in patients with curatively resected PDAC was associated with rapid development of distant metastasis; those who expressed either *SMAD4* or *KLF10* had a significantly higher chances of benefiting from adjuvant CRT (for patients with *KLF10* or *SMAD4* expression: GEM–CRT vs. GEM: PFS ∞ vs. 19.8 months; *p* = 0.026; OS 33 vs. 23 months; *p* = 0.12) [58]. The tryptophan catabolic enzyme, indoleamine 2,3 dioxygenase-2 (IDO2) has been demonstrated to promote pancreatic tumourigenesis in preclinical studies [59]. An IDO2-deficient genotype correlates with improved PFS for patients with PDAC who received adjuvant radiotherapy (39.0 ± 6.3 vs. 74.1 ± 6.4 months, *p* = 0.023). Analysis of metabolic profiles from patients with resectable PDAC receiving neoadjuvant therapy demonstrated a significant difference in choline metabolism between those responding favourably and unfavourably. Lower levels of choline and phosphocholine correlated with a low recurrence rate among patients with PDAC receiving neoadjuvant CRT [60]. Genomic profiling using targeted gene sequencing for radiotherapy response prediction was evaluated among 88 patients with cancer receiving local tumour irradiation. Alterations of DNA repair pathways and mutations of *CHEK2, MSH2 and NOTCH1* were associated with durable local control using radiotherapy [61]. A radiation sensitivity index (RSI) score for intrinsic tumour radiosensitivity derived from the expression of 10 specific genes (*HDAC1, PKCb, RelA, c-Abl, STAT1, AR, Cdk1, c-Jun, SUMO1*, and *IRF1*) and a linear regression algorithm modeled on the surviving fraction at 2 Gy (SF2) of 48 cancer cells were evaluated for 73 patients with PDAC receiving surgery with or without radiotherapy. Among high-risk patients, radiotherapy provided significantly improved survival among radio-sensitive patients compared with radio-resistant patients (*p* = 0.04). This difference was not observed among low-risk patients [62]. The RSI score was combined with the linear quadratic model to derive a genomic-adjusted radiation dose (GARD) by the same group of investigators to identify the optimum radiotherapy dose at a patient-specific molecular signature level. A high GARD value predicted a strong therapeutic effect of radiotherapy and greater time to first recurrence and OS. GARD independently predicted clinical outcomes for pancreatic cancer, and its use enabled the individualization of radiotherapy dose according to the tumour radiosensitivity [63, 64].

**Table 2:** Studies of potential tissue biomarker for PDAC patients considering radiotherapy

Study	Study type	Pt no.	PDAC stage	Tissue Origin	Treatment	biomarker	Endpoint	Conclusion	Significance
2011 Crane CH [52]	Pro-spective phase II	69	LA	Cytology	GEMOX + cetuximab + capecitabine-CRT	Smad4	Failure pattern	Pattern of progression may be predictable on the basis of Smad4 expression	intact Smad4 in 11/15 (73.3%) of local dominant recurrence. Smad4 loss in 10/14 (71.4%) distant dominant recurrence P = 0.016
2017 Shin SH [53]	Retro-spective	641	resectable	IHC	Adjuvant 5-FU/LV or GEM; 5-FU-CRT for R1 resection	Smad4	OS, recurrence	1.Inactivation Smad4 indicate metastasis 2.In expressed Smad4, local therapy contributes to improved survival	1. HR: 4.28 2. p = 0.002
2015 Whittle MC [54]	Retro-spective	88	resectable	IHC /ICGC	Chemotherapy with or without radiotherapy	Runx3	OS, relapse pattern	Low Runx3 benefit from radiotherapy	p < 0.018
2021 Pen SL [58]	Pro-spective phase III	111	resectable	IHC	Adjuvant GEM +/- GEM-CRT	Smad4, KLF10, Runx3	OS, RFS	Combining KLF10 and Smad4 may predict the benefits of adjuvant CRT in resected PDAC	High KLF10 or Smad4 (n = 55) had better local RFS (p = 0.026) and longer OS (p = 0.12) receiving adjuvant CRT than GEM alone.
2019 Nevler A [59]	Retro-spective	129	resected	DNA/ TCGA	With or without radiotherapy	Indoleamine 2,3 dioxygenase 2 (IDO2)	RFS	IDO2 inactivation associated with improved RFS in response to RT	p = 0.023
2022 Wada Y [60]	Retro-spective	88	resected	Frozen tissue	Resected with or without Neoadjuvant CRT	Choline metabolites	RFS	Reduced choline metabolites correlate with better RFS especially in NA-CRT group	Choline: P = 0.0022 (in NA-CRT: p = 0.028) Phospho-choline: p = 0.0086 (in NA-CRT p = 0.0037)
2015 Strom T [62]	Retro-spective	73	resectable	DNA	Adjuvant GEM/5FU ± RT(n = 61) No adjuvant therapy (n = 12)	10 specific genes (RSI score)	OS	Among clinical high risk irradiated patients, RSI low (radiosensitive) had significantly improved survival	High risk patient (R1, N1, postop CA19-9 > 90, n = 31) RSI low vs. RSI high OS:31.2 vs. 13.2 months, p = 0.04

Pt: patient; LA: locally advanced; GEMOX: gemcitabine + oxaliplatin; CRT: chemoradiotherapy; IHC: immunohistochemistry;5-FU/LV: 5-fluorouracil/leucovorin; ICGC: International Cancer Genome Consortium; RFS: recurrence-free survival; OS: overall survival; TCGA: the cancer genome atlas; NAT: neoadjuvant therapy; RSI: radiation sensitivity index

Several peripheral blood biomarkers have been demonstrated to determine survival or therapeutic response in PDAC (Table 3). Absolute monocyte count during CRT and changes in the lymphocyte-to-monocyte ratio correlated with OS and PFS among patients with LAPC treated with CRT [65]. The baseline neutrophil-to-lymphocyte ratio (NLR) and NLR dynamics during neoadjuvant chemotherapy were independently associated with pathologic response in resectable PDAC [66]. Despite not being specific to a cancerous condition and a lack of expression in 5 -10% of patients, CA19-9 is the most used tumour marker for monitoring therapy for PDAC. A decrease in the CA19-9 level after neoadjuvant therapy is correlated with improved OS and pathologic major response in PDAC [67–69]. We analyzed CA19-9 change during adjuvant chemotherapy among 125 patients with resected PDAC with or without adjuvant radiation. Significant correlations of CA19-9 response with initial failure at distant sites and OS were identified. However, neither postoperative CA19-9 level nor CA19-9 response were helpful in identifying patients who may experience a survival benefit from additional adjuvant CRT [70]. A retrospective analysis reported that a high level of carcinoembryonic antigen but not CA19-9 before neoadjuvant CRT was the most significant predictor of poor survival after surgery for PDAC [71]. Regarding other circulating biomarkers, baseline CC motif chemokine ligand 5 (CCL5) was identified as an independent prognostic biomarker for OS in patients with LAPC in the Selective Chemoradiation in Advanced Localised Pancreatic Cancer (SCALOP) study, which evaluated induction GEM-Cape and CRT [72]. A correlation between CCL5 levels and failure patterns was not identified. Increasing evidence indicates that microRNAs (miRNAs) may serve as diagnostic, predictive and prognostic biomarkers in various cancer entities, including PDAC. The expression of miRNAs was correlated with pancreatic cancer progression and radio-resistance [73]. A four-miRNA molecular signature (miR-29c, miR-125a, miR-155, and mR-200b) was developed to predict risk of locoregional recurrence and OS after PDAC resection. Using the miRNA risk score has potential for identifying patients with PDAC who are most likely to benefit from postoperative CRT [74]. Circulating tumor DNA (ctDNA) is released into the peripheral blood stream during cell death. The presence of ctDNA in patients with PDAC after neoadjuvant therapy indicates recurrence and poor survival [75, 76]. Circulating tumour cells that enter peripheral blood are thought to contribute to metastatic disease with worse survival [77]. In an analysis of the Surveillance, Epidemiology, and End Results database, patients with PDAC with a tumour location over the pancreatic head, stage II/III cancer, T4 cancer, N1 cancer, regional resection, or lymphadenectomy of ≥ 4 lymph nodes were demonstrated to benefit from adjuvant radiotherapy [78, 79]. Several studies have revealed that a combined analysis of radiomic features, clinical parameters, pathology score, and tissue/serum biomarkers improves the prognostic power of clinical outcomes in PDAC [32, 80].

**Table 3:** Studies of potential peripheral blood biomarkers for PDAC patients considering radiotherapy

Study	Study type	Pt No.	PDAC stage	Timing of collection	Treatment	Biomarker	Endpoint	Conclusion	Significance
2021 Perri G [69]	Retro-spective	290	Resected	Serum after neoadjuvant therapy	FOLFIRINOX, or GEM + Nab, +/- CRT	Post treatment CA19-9	pMR (< 5% residual cancer cells)	Post-treatment CA19-9 level independently associated with pMR.	CA19-9 of pMR vs. others: 17 vs. 30 U/mL (P < 0.01)
2023 Chiu YF [70]	Pro-spective	125	Resectable	Serum during adjuvant GEM	1. GEM 2. GEM + Gem-CRT	CA19-9 response	OS	CA19-9 response to initial adjuvant therapy predict survival and failure pattern after resection.	CA19-9 response to OS: p = 0.0008 CA199 response to distant failure: p = 0.023
2022 Kato H [71]	Retro-spective	72	LAPC	Serum after NAC + RT	Gem + S1 + RT	CEA	OS	LAPC with CEA > 7.2 ng/mL should be recognized as systemic disease	CEA > 7.2ng/mL (n = 15) vs. CEA < 7.2ng/ml (n = 57) (8 vs. 24 months, p < 0.00001)
2021 Willenbrock F [72]	Pro-spective randomized phase II	60	LAPC	Baseline serum	GEM-Cape x 3: 1.GEM-Cape + Gem-CRT 2.GEM-Cape + Cape based-CRT	CCL5	OS	Low CCL5 significantly associated with improve OS	CCL5 low vs. high to OS: 18.5 vs. 11.3 months, P = 0.037
2017 Giacomelli [65]	Retro-spective	57	NAT and resected LAPC	Blood before, during and after CRT	Before (TP1), during(TP2), and at the end(TP3) of CRT	LMR	OS PFS	1. Absolute monocyte counts during CRT associated with PFS 2. LMR change (TP3/TP2) > 0.32 predict OS	1. P = 0.04 2. P < 0.0001
2022 De Castro Silva [66]	Retro-spective	94	NAC and resected PDAC	Blood before and after NAC	Baseline, after NAT	NLR	Pathologic Response, PFS, OS	1. Baseline NLR and ∆NLR associated with pathologic response 2. NLR score correlated with PFS and OS	1. p < 0.001, p = 0.002 2. P = 0.006, p = 0.002
2020 Wolfe AR [74]	Retro-spective	88	Resected	Baseline RNA	With or without chemotherapy; no radiotherapy	4-miRNAs signature	Local recurrence, OS	The 4-miRNA signature has the potential to select patients most likely benefit from CRT	Local recurrence: P = 0.001 OS: p = 0.034

GEM: gemcitabine; Nab: nab-paclitaxel; FOLFIRINOX: 5-fluorouracil, oxaliplatin, irinotecan, leucovorin; Cape: capecitabine; pMR: pathologic major response; LAPC: locally advanced pancreatic cancer; LMR: lymphocyte to monocyte ratio; NAC: neoadjuvant chemotherapy, NAT: neoadjuvant therapy; RT: radiotherapy; CRT: chemoradiotherapy; LMR: lymphocyte to monocyte ratio; NLR: neutrophil to lymphocyte ratio; NLR score = baseline NLR+∆ NLR; ∆NLR = pre-surgery-pre-chemotherapy NLR

## Conclusions

Despite progress in surgical techniques and systemic therapy, the survival outcomes of patients with PDAC remain unsatisfactory. Radiotherapy was a central component of treatment for PDAC. The value of CRT to PDAC has been questioned because of conflicting results of clinical trials. Most studies have been criticised for low patient numbers, poor study design, inappropriate radiation doses or split-course regimens, and poor adherence to the radiation protocol [81–83]. However, several prospective trials have demonstrated the efficacy of modern radiation therapy, with an elevated dosage and reduced toxicity to the small bowel, exhibiting a satisfactory safety profile, local control, and prolonged survival for localised PDAC [25, 26]. In addition to the technical improvement of radiotherapy, the development of radiogenomics and the biology of radiotherapy for PDAC may help to optimise the integration of radiotherapy in multimodality PDAC treatment strategies. Because distant metastases are more effectively controlled through modern systemic therapy, local control of the primary site is increasingly critical for patients with PDAC with extended survival [23]. Advances in radiomic, tissue, or peripheral biomarkers may enable superior stratification of patients’ metastatic potential and prediction of those who would most likely benefit from enhanced locoregional therapy. However, studies evaluating the role of potential biomarkers have mostly been retrospective and have demonstrated correlations with survival but not failure patterns. Multi-institutional prospective clinical trials that validate candidate biomarkers in patients with PDAC receiving up-to-date systemic chemotherapy with or without modern radiotherapy are urgently required.

The role of radiotherapy in the curative treatment of PDAC remains unclear. In designing future clinical trials, the exclusion of patients with early distant progression by extended systemic therapy (≥ 4 months) and predictive biomarkers is reasonable. Local control using radiotherapy may yield a survival benefit, especially among patients with PDAC without early distant metastasis.

## Data Availability

Data sharing not applicable to this article as no datasets were generated or analyzed during the study.

## List of abbreviations

PDAC: pancreatic adenocarcinoma
CRT: chemoradiotherapy
LAPC: locally advanced pancreatic cancer
FOLFIRINOX: leucovorin calcium (folinic acid), fluorouracil, irinotecan hydrochloride, oxaliplatin
GEM-Nab: gemcitabine plus nab-paclitaxel
5FU: 5-fluorouracil
OS: overall survival
GEM-Cape: gemcitabine plus capecitabine
SBRT: stereotactic body radiotherapy
MRI: magnetic resonance imaging
PFS: progression-free survival
nAUC: normalized area under the enhancement curve
LAI: local advancement Index
FDG-PET: fluoro-deoxygluocse positron emission tomography
KLF10: krüppel-like factor 10
IDO2: indoleamine 2,3 dioxygenase-2
RSI: radiation sensitivity index
SF2: surviving fraction at 2 Gy
GARD: genomic-adjusted radiation dose
LMR: lymphocyte to monocyte ratio
NLR: neutrophil to lymphocyte ratio
CCL5: c-c motif chemokine ligand 5
CTCs: circulating tumor cells
SEER: surveillance, epidemiology, and end results
